# Decreased BIRC5-206 promotes epithelial–mesenchymal transition in nasopharyngeal carcinoma through sponging miR-145-5p

**DOI:** 10.1515/med-2024-1007

**Published:** 2024-09-17

**Authors:** Weihua Xu, Junjie Hu, Zhichao Ma, Wanyi Feng, Wei Gong, Shengmiao Fu, Xinping Chen

**Affiliations:** Department of Medical Laboratory, Hainan Cancer Hospital, Affiliated Cancer Hospital of Hainan Medical University, Hainan Tropical Cancer Research Institute, Haikou, Hainan, 570312, China; Hainan Lvtou Medical Laboratory Center, Haikou, Hainan, 570206, China; School of Life Sciences, Hainan University, Haikou, Hainan, 570228, China; Central Laboratory, Hainan General Hospital, Hainan Hospital Affiliated to the Hainan Medical College, No. 19 Xiuhua Road, Xiuying District, Haikou, Hainan, 570311, China; Department of Medical Laboratory, Hainan Cancer Hospital, Affiliated Cancer Hospital of Hainan Medical University, Hainan Tropical Cancer Research Institute, No. 6, Changbin West 4th Street, Xiuying District, Haikou, Hainan, 570312, China; Hainan Lvtou Medical Laboratory Center, No. 16 Jinyu East Road, Longhua District, Haikou, Hainan, 570206, China

**Keywords:** nasopharyngeal carcinoma, BIRC5-206, epithelial–mesenchymal transition, miR-145-5p, CD40

## Abstract

Metastasis significantly contributes to the poor prognosis of advanced nasopharyngeal carcinoma (NPC). Our prior studies have demonstrated a decrease in BIRC5-206 expression in NPC, which promotes disease progression. However, the role of BIRC5-206 in the invasion and metastasis of NPC has not been fully elucidated. In this study, our objective was to explore the biological function and underlying mechanisms of BIRC5-206 in NPC. Additionally, we established an NPC mouse model of lung invasiveness using C666 cells to assess the impact of BIRC5-206 on NPC metastasis. Our results revealed that silencing BIRC5-206 inhibited apoptosis and enhanced the invasion of NPC cells, whereas its overexpression reversed these effects. Moreover, decreased BIRC5-206 expression significantly increased N-cadherin and Vimentin expression while reducing E-cadherin and occludin levels, both *in vivo* and *in vitro*. Additionally, silencing BIRC5-206 markedly augmented the formation of invasive foci in lung tissues. Rescue experiments further confirmed that decreased BIRC5-206 expression facilitates NPC metastasis via modulation of the miR-145-5p/CD40 signaling pathway. In summary, our study suggests that BIRC5-206 may serve as a potential prognostic biomarker and therapeutic target in the diagnosis and treatment of NPC.

## 1 Introduction

Nasopharyngeal carcinoma (NPC) is a prevalently malignant tumor in otolaryngology and head and neck surgery [1]. In 2020, of the estimated 133,354 newly diagnosed cases of NPC and 80,008 associated deaths, the majority are expected to occur in South-East Asia [2]. Notably, China accounts for approximately 80% of global NPC cases, with around 80% of these concentrated in six provinces of South China [3]. The incidence of NPC in these regions is alarmingly high, with annual rates reaching 30–50 cases per 100,000 individuals [4]. Moreover, an increasing trend in the incidence of NPC is anticipated annually in this region. Metastasis remains the leading cause of mortality in patients diagnosed with NPC [5], with the liver and lungs being the most common metastasis sites in NPC cases [4]. Consequently, investigating the molecular mechanisms underlying NPC metastasis and developing new biomarkers for predicting the development of primary nasopharyngeal cancer are of critical importance. Such advancements could significantly enhance the prevention and treatment strategies for NPC.

Research indicates that epithelial–mesenchymal transition (EMT) plays a pivotal role in enhancing the migratory and invasive capabilities of cancer cells [6]. EMT involves the transformation of epithelial cells into a mesenchymal phenotype, marked by the loss of cell–cell junctions and apical–basal polarity, and the acquisition of migratory and invasive characteristics [7]. Molecular markers indicative of EMT include the downregulation of epithelial markers like E-cadherin and occludin and the upregulation of mesenchymal markers like N-cadherin, vimentin, and fibronectin [8–10]. Moreover, EMT is associated with various cancer-related features, including resistance to apoptosis, the acquisition of stem cell-like traits, and resistance to chemotherapy [11–13].

BIRC5, prominently overexpressed in virtually all human malignancies, is strongly associated with a spectrum of aggressive tumor characteristics [14,15]. Elevated expression of Survivin (encoded by the BIRC5 gene) correlates with increased tumor aggressiveness, poor therapeutic response, unfavorable prognosis, heightened risk of relapse, and diminished survival rates [15–18]. Notably, in stage IV lung cancer patients, BIRC5 levels were found to be significantly higher compared to those in patients without distant metastases, with concentrations escalating in correlation to the number of affected metastatic organ sites [19]. In breast carcinoma, a marked increase in BIRC5 mRNA levels was observed, and its overexpression was linked to a deterioration in both overall and relapse-free survival rates [20]. Furthermore, BIRC5 expression in gastric cancer demonstrated significant associations with gross morphology, depth of invasion, presence of distant metastases, tumor necrosis, metastatic stage, and vascular invasion [21].

Survivin, encoded by the BIRC5 gene, belongs to the inhibitor of apoptosis protein (IAP) family [22]. Unlike other IAP proteins, Survivin is typically expressed during embryonic and fetal development but remains undetectable in normal adult tissues [20]. Survivin gene spans four exons and three introns, encompassing 14,796 nucleotides, and is located on chromosome 17 (17q25) [22]. BIRC5 encodes the wild-type Survivin (WT) isoform, comprising four exons and 142 amino acids (NM_001168.3). Additionally, four known splicing variants of Survivin have been identified: ΔEx3 (Survivin with an exon 3 deletion, 137 amino acids, NM_001012270.2), 2B (Survivin with additional exons, 165 amino acids, NM_001012271.2), 3B (comprising five exons, 120 amino acids, XR_243654.5), and 2α (consisting of two exons, 74 amino acids, XR_934452.3) [20]. The functional attributes of each Survivin variant are influenced by their specific structural characteristics, including the presence or absence of particular domains, which dictate their role in apoptotic or tumorigenic pathway [20].

Survivin proteins are notably overexpressed in NPC and are associated with adverse outcomes, including poor overall survival, lymph node metastasis, local recurrence, distant metastasis, and higher clinical stages [23,24]. In our prior research, we identified a significant downregulation of BIRC5-206 (ENST00000589892.1), a non-coding splice variant of Survivin, which spans 747 base pairs and functions as a non-coding RNA. This downregulation was found to contribute to NPC progression by promoting the transformation of NPC cells into cancer stem cells [25]. However, the specific role and function of BIRC5-206 in NPC metastasis have not been fully determined. Therefore, the primary aim of our current study is to delve into the specific role and molecular mechanisms underlying BIRC5-206 in the invasion and metastasis of NPC. This research is intended to enhance our understanding of NPC pathogenesis and potentially inform the development of more effective therapeutic approaches.

## 2 Methods

### 2.1 Cell culture and vector construction and lentiviral infection

The human NPC cell lines C666 and HNE3 were kindly provided by the Nasopharyngeal Cancer Research Laboratory at Guangxi Medical University. These cells were cultured in RPMI 1640 medium (cat. no. 11875119) supplemented with 10% fetal bovine serum (FBS, cat. no. 16140071) and 1% penicillin/streptomycin (cat. no. 15140122) from Gibco (Thermo Fisher Scientific, Inc.). The culture conditions were atmospheric oxygen tension (∼21%) and 5% CO_2_ at 37°C.

Human BIRC5-206 sequences from the Ensembl databases (www.ensembl.org, Ensembl gene: ENST00000589892.1), synthesized by GeneChem Co., Ltd (Shanghai, China), were subcloned into a GV358 vector (Component Order: Ubi-MCS-3FLAG-SV40-EGFP-IRES-puromycin) to generate BIRC5-206-overexpressing plasmids. 293T human embryonic kidney cells (Chinese Academy of Medical Science, Beijing, China) were transfected with a mixture of plasmids, including viral packaging plasmids psPAX2 (7.5 µg), envelope plasmid pMD2.G (2.5 µg), and the BIRC5-206 expression plasmid (GV358- BIRC5-206, 25 µg) or the control plasmid (GV358, 25 µg) via Lipofectamine 2000 (Invitrogen; Thermo Fisher Scientific, Inc.) based on the manufacturer’s protocol. Lentivirus supernatant was collected and filtered through a 0.45 μM filter 48 h post-transfection.

C666 and HNE3 cells were seeded in six-well plates at a density of 1  ×  10^5^ cells/well and cultured at 5% CO_2_ and 37°C. The cells were transfected the next day at approximately 70% confluency. Transfection was conducted using Lipofectamine^®^ 2000 (Invitrogen; Thermo Fisher Scientific, Inc.) at MOI  =  10 for negative control and MOI  =  20 for lentivirus-BIRC5-206. Post-transfection, C666 and HNE3 cells were incubated at 37°C for 8 h, after which the medium was replaced with fresh complete medium. Puromycin (2 μg/mL) was used to select for puromycin-positive cells 48 h after transfection. The expression of BIRC5-206 was subsequently assessed using quantitative PCR.

A lentivirus carrying BIRC5-206 shRNA was constructed by Genechem Co., Ltd (Shanghai, China) for transfection into C666 and HNE3 cells. The shRNA sequences used were as follows: control shRNA sequence (NC), 5′-TTCTCCGAACGTGTCACGT-3′; BIRC5-206 shRNA-1, 5′-AAGTATGTTCACTATGAAA-3′ (KD1); BIRC5-206 shRNA-2, 5′-CAAGGAACAATCCATTGTT-3′ (KD2). These sequences were cloned into the lentiviral GV493 vectors (Component Order: hU6-MCS-CBh-gcGFPIRES-puromycin). The LV-BIRC5-206-shRNA was transfected into C666 and HNE3 cells upon reaching 60% confluence. Puromycin (2 μg/mL) was used for selection and was removed after 72 h.

### 2.2 Apoptosis analysis

Apoptosis in C666 and HNE3 cells was assessed using Annexin V and propidium iodide (PI) staining (cat. no. V13241; Thermo Fisher Scientific). The cells were rinsed twice with PBS, centrifuged at 1,000 rpm for 5 min, and then resuspended in 195 μL of Annexin V-FITC/PI binding buffer. To this suspension, Annexin V-FITC (5 μL) and PI (10 μL) were added. Then, the cells were incubated in the dark at room temperature for 40 min. Apoptosis was analyzed using a BD LSR II flow cytometer. For each sample, a total of ten thousand events were collected, and the data were analyzed using CXP analysis software (Beckman-Coulter, Fullerton, CA, USA).

### 2.3 Cell invasion assay

To evaluate the invasive potential of C666 and HNE3 cells post-transfection with BIRC5-206 lentivirus, a Transwell assay was conducted. Transwell chambers (cat. no. 3402, Corning Life Sciences) were pre-coated with Matrigel (cat. no. 356234; Corning Life Sciences) for 6 h at 37°C. The C666 and HNE3 cells were seeded in the upper compartment of the Transwell at a density of 6 × 10^3^ cells/well. In the lower chamber, 150 μL of serum-free medium was added, while the upper chamber received 600 μL of RPMI 1640 medium (cat. no. 11875119, Gibco). Following 48 h of incubation at 37°C, cells on the basolateral side of the chamber were washed twice with PBS and stained with 1% crystal violet for 30 min at room temperature. After two additional washes with PBS, the stained C666 and HNE3 cells were examined and imaged using a light microscope at 200× magnification (Olympus cX2; Olympus Corporation).

### 2.4 Western blot

Proteins were extracted from C666 and HNE3 cells using RIPA lysis buffer (cat. no. HY-K1001; MedChemExpress), which contains 1% protease/phosphatase inhibitor cocktail (cat. no. 5872, Cell Signaling). The concentration of the extracted protein was determined using the BCA Protein Assay kit (cat. no. 23225; Thermo Fisher Scientific, Inc.). Isolated proteins (5 μg) were then mixed with 5× SDS-PAGE protein loading sample buffer (cat. no. 20315ES05, Yeasen Biotechnology Co., Ltd, China) and then loaded onto 12% SDS-acrylamide gels for separation by electrophoresis. Post-separation, the proteins were then transferred onto PVDF membranes and blocked with 5% non-fat milk. The membranes were incubated overnight at 4°C with primary antibodies, including anti-CD40 (1:1,000; cat. no. ab252557; Abcam), anti-N-cadherin (1:1,000; cat. no. ab76011; Abcam), anti-Vimentin (1:1,000; cat. no. ab92547; Abcam), anti-E-cadherin (1:1,000; cat. no. ab40772; Abcam), anti-occludin (1:1,000; cat. no. ab216327; Abcam), and anti-GAPDH (1:10,000; cat. no. ab181602; Abcam). Following the primary antibody incubation, the membranes were washed and then incubated with horseradish peroxidase-conjugated goat anti-rabbit IgG secondary antibody (1:10,000; cat. no. ab6721; Abcam) for 1 h at room temperature. The protein bands were visualized using ECL western blot detection reagents (Thermo Fisher Scientific, Inc.), and their intensities were quantified using ImageJ software (Version 1.5.3, NIH ImageJ system).

### 2.5 Lung invasiveness assay in a nude mouse model

BALB/c mice, 6 weeks of age, were acquired from Beijing HFK Bioscience Co., Ltd. The mice were housed in a specific pathogen-free facility under controlled environmental conditions, with the temperature maintained at 23 ± 2℃. The mice had free access to food and water and were subjected to a 12-h light/dark cycle for 1 week to acclimatize. In the experiment, the mice received an intravenous injection via the lateral tail vein with a suspension of either NC (negative control) or BIRC5-206 knockdown C666 cells (4 × 10^5^ cells/mouse) in 0.1 mL of PBS. After 6 weeks, the mice were euthanized, and their lungs were extracted and photographed with a ruler for size measurement. All animal procedures were performed in accordance with the guidelines for experimental animal care and use.

### 2.6 Hematoxylin and eosin (HE) staining and immunohistochemistry (IHC)

For histological examination, mouse lung tissues were fixed with 4% paraformaldehyde at room temperature for 24 h and subsequently washed with 70% alcohol. The tissues were then embedded in paraffin and sectioned at a thickness of 5 µm. The sections were stained using a Hematoxylin and Eosin Staining Kit (cat. no. C0105M; Beyotime) following the manufacturer’s instructions. Digital images were captured at a magnification of ×20 using a DP71 digital microscope camera attached to an Olympus microscope.

For IHC staining, the dewaxed, rehydrated tissue sections underwent antigen retrieval and blocking of endogenous peroxidase activity as per the manufacturer’s protocols. Antibodies against N-cadherin (1:200; cat. no. ab76011; Abcam), Vimentin (1:200; cat. no. ab92547; Abcam), E-cadherin (1:200; cat. no. ab40772; Abcam), and occludin (1:200; cat. no. ab216327; Abcam) were applied to the sections and incubated overnight at 4°C. This was followed by incubation with a biotinylated secondary goat anti-rabbit antibody at room temperature for 30 min. 3,3′-Diaminobenzidine was used as the chromogen, with hematoxylin as the counterstain. The stained slides were digitized using the Aperio ScanScope CS Slide Scanner (Aperio Technologies). Three randomly selected fields of view per section were analyzed to calculate the mean and standard error of the mean of cells exhibiting positive staining.

### 2.7 RNA fluorescence *in situ* hybridization (FISH)

An RNA FISH assay was performed using the Ribo lncRNA FISH Probe Kit (cat. no. C10920, Ribo, China) following the manufacturer’s instructions. Briefly, C666 and HNE3 cells were cultured and fixed with 4% paraformaldehyde at room temperature. Following thorough washing, the cells were incubated overnight with hybridization solutions containing the digoxin-labeled BIRC5-206 probe (GAGGCTGCAGTGAGTTGTGACCGCACCGCTGCACTCCAGC). Subsequently, the cell nuclei were stained with DAPI for 15 min. The cells were then visualized and images were captured using a fluorescence microscope (Olympus, Japan).

### 2.8 Target gene prediction

The Bibiserv database (https://bibiserv.cebitec.uni-bielefeld.de/rnahybrid/) is used to predict the target miRNAs of BIRC5-206. The target gene prediction database Targetscan (version 6.2; http://www.targetscan.org) was used to identify potential targets of miR-145-5p.

### 2.9 Luciferase activity assay and RNA immunoprecipitation (RIP)

The 3′-UTR of BIRC5-206 or CD40 was amplified and subcloned into the pMIR luciferase reporter vector. Similarly, mutant fragments of BIRC5-206 and CD40 were inserted into the pMIR luciferase reporter vector. The luciferase report vector (carrying BIRC5-206-WT/MUT or CD40-WT/MUT) was transfected into 293T cells with either miR-145-5p or miR-NC. The relative luciferase activity in each group was determined at 48 h post-transfection using a luciferase assay kit (Pharmingen, San Diego, CA, USA).

A RIP assay was conducted using the RIP Kit (cat. no. Bes5101, BersinBio, China). C666 cells were washed with pre-chilled PBS and lysed in RIP buffer at 4°C for 30 min. Magnetic beads conjugated with human anti-Ago2 antibodies (cat. no. APREST91863, PrEST Antigen) or normal mouse immunoglobulin G (IgG; cat. no. 2729; Cell Signaling Technology, USA) were employed to capture the RNAs. RNA was then extracted from the precipitates using TRIzol reagent (cat. no. 9108, Takara Biotechnology, China). The expression levels of BIRC5-206 and miR-145-5p were quantified using RT-PCR. The primers for the target genes are as follows: miR-145-5p RT: 5′-GTCGTATCCAGTGCAGGGTCCGAGGTATTCGCACTGGATACGACAGGGAT-3′; miR-145-5p forward 5′-GCCGAGGTCCAGTTTTCCCAGGA-3′ and reverse 5′-CTCAACTGGTGTCGTGGAGTCGGCAATTCAGTTGAG-3′; BIRC5-206 forward 5′-ATGTGGGGGGAGATGTCCAC-3′ and reverse 5′-GCAGTCCACTCCCCACGG-3′.

### 2.10 miR-145-5p mimic and inhibitor transfections

The miR-145-5p mimic (5′-GUCCAGUUUUCCCAGGAAUCCCU-3′), mimic negative control (Mimic-NC, 5′-CGCGAGUUAACGGACCAUACGGU-3′), miR-145-5p inhibitor (5′-AGGGAUUCCUGGGAAAACUGGAC-3′), and inhibitor negative control (Inhibitor-NC, 5′-CAGUACUUUUGUGUAGUACAA-3′) were synthesized by Shanghai GenePharma Company. The miR-145-5p mimic, mimic NC, miR-145-5p inhibitor, and inhibitor NC were delivered into C666 cells using Oligofectamine™ according to the manufacturer’s instructions.

### 2.11 Quantitative real-time polymerase chain reaction (qPCR)

Total RNA was extracted from C666 and HNE3 cells using TRIzol reagent (cat. no. 9108, Takara Biotechnology, China). The concentration of RNA was measured using an ND-2000 Spectrophotometer (Thermo Fisher Scientific, USA). qPCR was performed using a 7300 Real-Time PCR System (Applied Biosystems). The PCR amplification protocol included an initial denaturation at 95°C for 5 min, followed by 40 cycles of denaturation at 95°C for 5 s and annealing/extension at 61°C for 30 s. The qPCR results were analyzed using the 2^−ΔΔCt^ method [26]. The expression levels of CD40 and BIRC5-206/miR-145-5p were normalized to the reference genes GAPDH and U6, respectively. The primers for the target genes were as follows: BIRC5-206 forward 5′-GAGGCTGGCTTCATCCACTG-3′ and reverse 5′-TGGTTTCCTTTGCATGGGGT-3′; miR-145-5p forward: 5′-ACACTCCAGCTGGGGTCCAGTTTTCCCAGGA-3′, reverse: 5′-GTCCAGTTTTCCCAGGAATCCCTAGGGATTC-3′; U6 forward: 5′-CTCGCTTCGGCAGCACA-3′, reverse: 5′-AACGCTTCACGAATTTGCGT-3′. CD40 forward 5′-CTGATGTTGTCTGTGGTCCCC-3′ and reverse 5′-TGGCCACCTTTTTGATAAAGACC-3′; and GAPDH forward 5′-CACCATCTTCCAGGAGCGAG-3′ and reverse 5′-AAATGAGCCCCAGCCTTCTC-3′.

### 2.12 Statistical analysis

Statistical analysis was carried out using GraphPad Prism software (version 8.0; California, USA). Each experiment was repeated three times, and the data are presented as mean ± standard deviation. Comparisons between two groups were made using Student’s *t*-test, while one-way analysis of variance test was employed for multiple comparisons, followed by Duncan’s post hoc test. A *p*-value of less than 0.05 was considered to indicate statistical significance.


**Ethical approval:** The experimental protocol was approved by the Ethics Committee of Hainan General Hospital (ethical approval number: YYFY-LL-2023-121).

## 3 Results

### 3.1 Decreased BIRC5-206 contributed to the invasion of NPC cells

**Figure 1:**
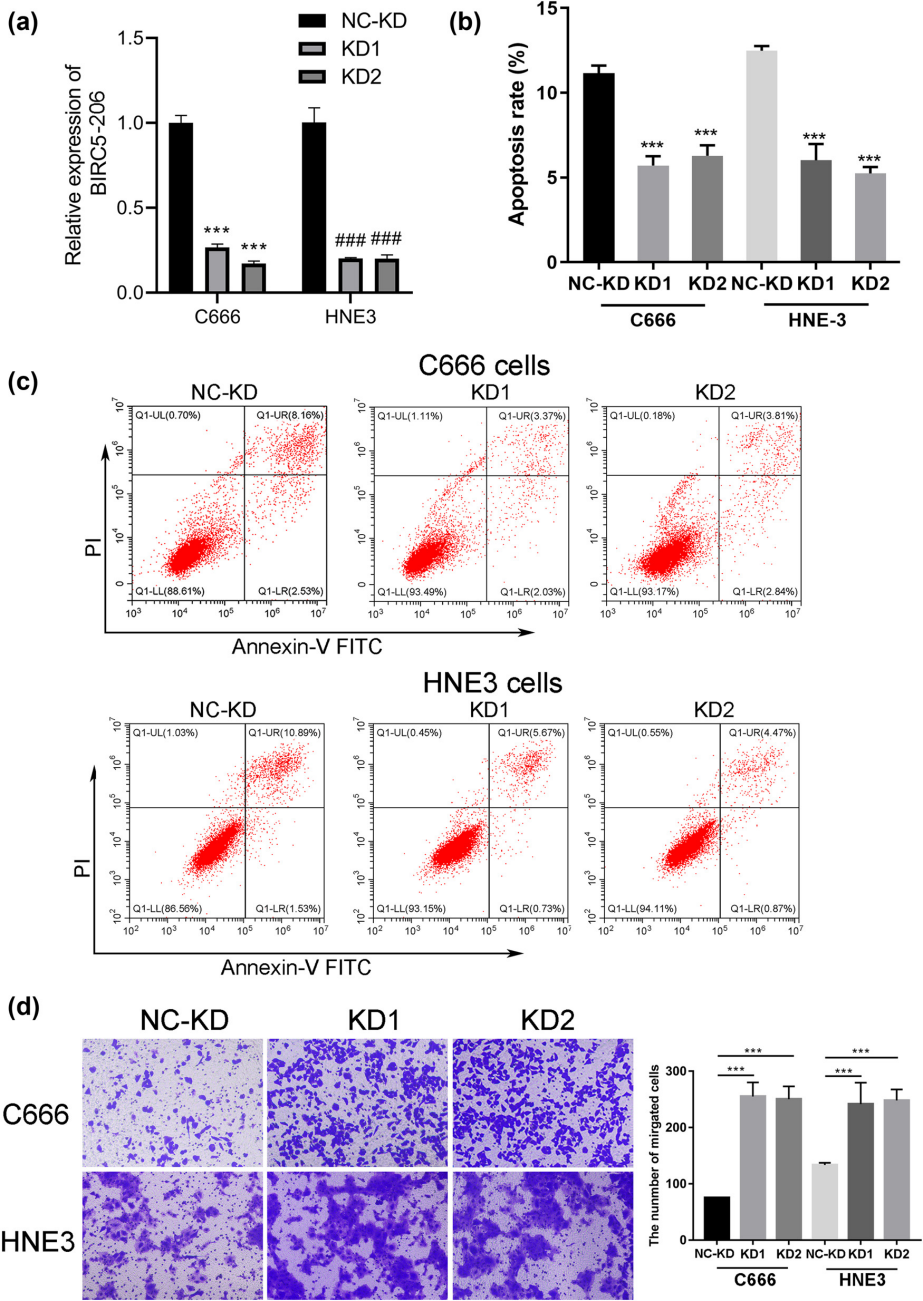
Decreased BIRC5-206 enhances the invasion of NPC cells. Human NPC cell lines C666 and HNE3 were transfected with BIRC5-206 shRNA. (a) The mRNA level of BIRC5-206 in C666 and HNE3 cells was detected by qPCR. (b) and (c) The apoptosis rate of C666 and HNE3 cells was evaluated using PI staining detected by Flow cytometry. (d) The invasion ability of C666 and HNE3 cells following BIRC5-206 silencing was assessed using a Transwell invasion assay. Results are expressed as means ± standard deviation (*n* = 3, ***p < 0.001). Abbreviation: NC-KD, negative control group; KD1, BIRC5-206 knockdown by shRNA-1; KD2, BIRC5-206 knockdown by shRNA-2.

**Figure 2:**
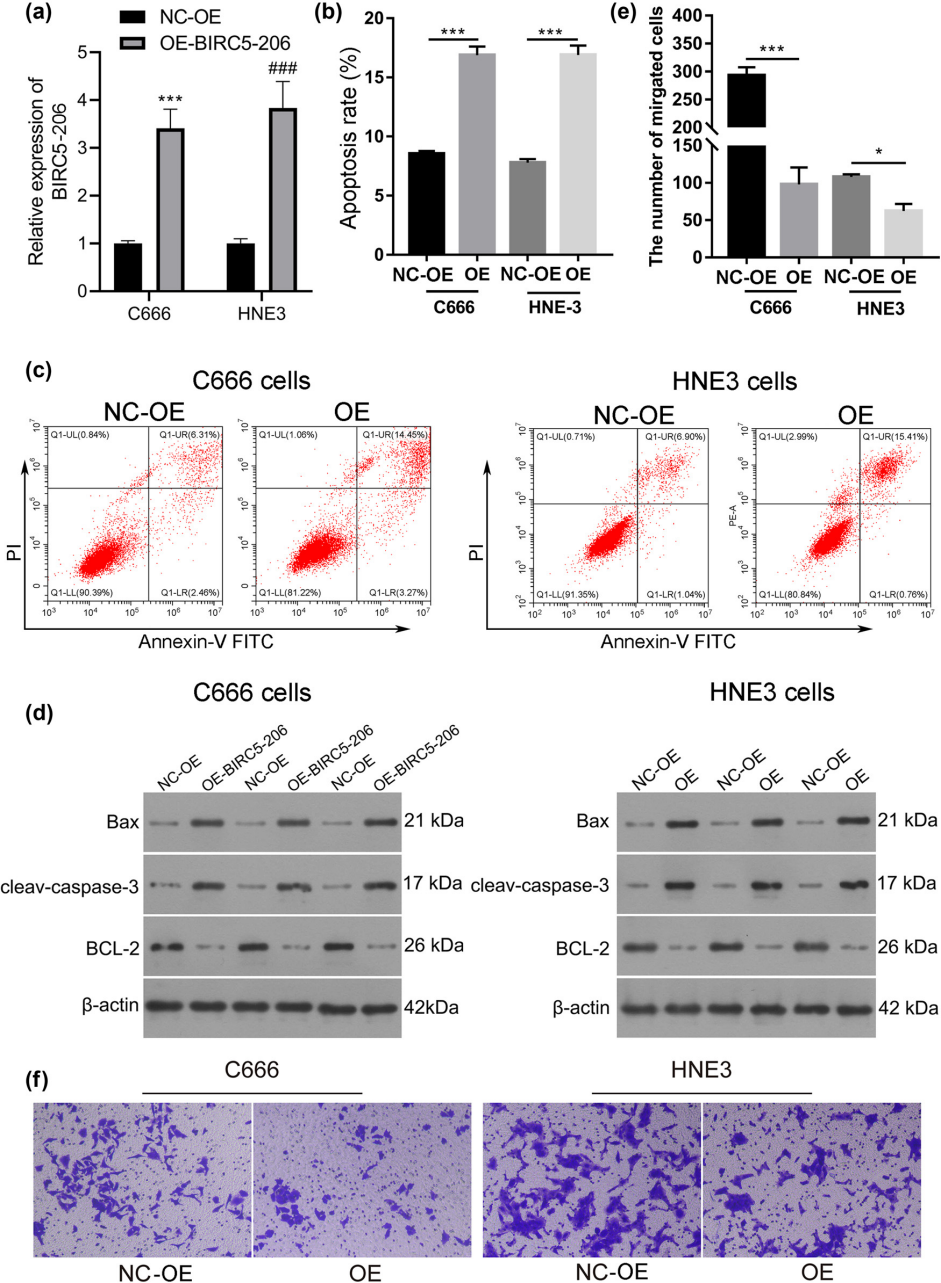
Overexpression of BIRC5-206 inhibits invasion of NPC cells. C666 and HNE3 cells were transfected with BIRC5-206 overexpression lentivirus. (a) The mRNA level of BIRC5-206 in C666 and HNE3 cells was detected by qPCR. (b) and (c) The apoptosis rate of C666 and HNE3 cells was detected by Flow cytometry. (d) The protein level of Bax, cleav-caspase-3 and BCL-2 in C666 and HNE3 cells was detected by Western blot. (e) and (f) The invasion ability of C666 and HNE3 cells following BIRC5-206 overexpression was assessed using a Transwell invasion assay. Results are expressed as means ± standard deviation (*n* = 3, *p < 0.05, ***p < 0.001).

To explore the potential role of BIRC5-206 in NPC, we performed experiments involving the knockdown and overexpression of BIRC5-206 in NPC cell lines C666 and HNE3. We observed a significant decrease in the mRNA levels of BIRC5-206 in the knockdown groups, confirming the successful downregulation of BIRC5-206 in C666 and HNE3 cells (*p* < 0.001, Figure 1a). Flow cytometry analysis revealed a marked reduction in the apoptosis rate of these cells following BIRC5-206 knockdown (*p* < 0.001, Figure 1b and c). Additionally, BIRC5-206 knockdown significantly enhanced the invasion ability of both C666 and HNE3 cells (*p* < 0.001, Figure 1d). In contrast, overexpressing BIRC5-206 produced opposite effects. The mRNA levels of BIRC5-206 were significantly increased in the overexpression groups, indicating successful upregulation of BIRC5-206 in C666 and HNE3 cells (*p* < 0.001, Figure 2a). Flow cytometry analysis showed a significant increase in the apoptosis rate of these cells following BIRC5-206 overexpression (*p* < 0.001, Figure 2b and c). Notably, heightened levels of BIRC5-206 resulted in the upregulation of the pro-apoptotic gene Bax and cleaved caspase-3, while concurrently suppressing the expression of the anti-apoptotic gene BCL-2 in C666 and HNE3 cells (Figure 2d). Furthermore, overexpression of BIRC5-206 substantially reduced the invasion ability of C666 and HNE3 cells (*p* < 0.05, *p* < 0.001, Figure 2e and f).

### 3.2 BIRC5-206 knockdown promoted EMT *in vitro*

**Figure 3:**
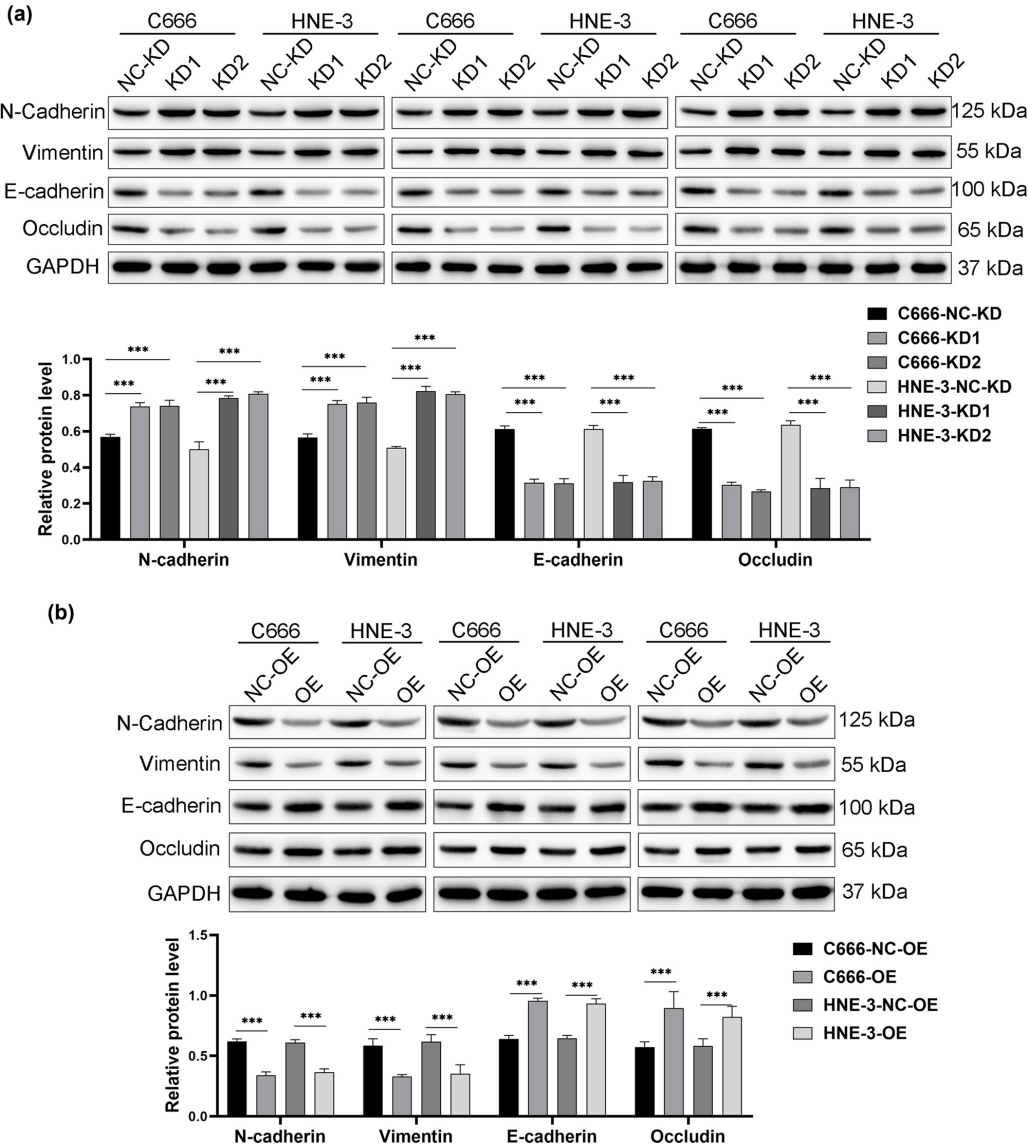
Decreased BIRC5-206 promotes EMT progression in NPC cells. (a) The protein levels of N-cadherin, Vimentin, E-cadherin, and occludin in BIRC5-206 knockdown C666 and HNE3 cells were detected by Western blot. (b) The protein levels of N-cadherin, Vimentin, E-cadherin, and occludin in BIRC5-206 overexpression C666 and HNE3 cells were detected by Western blot. Grayscale analysis of the Western blot results was performed using ImageJ software. Results are expressed as means ± standard deviation (*n* = 3, ***p < 0.001).

In this study, we explored the effects of BIRC5-206 knockdown on EMT in both *in vitro* and *in vivo* settings. Our results show that BIRC5-206 knockdown significantly increased the protein levels of N-cadherin and Vimentin, while simultaneously decreasing the protein levels of E-cadherin and occludin in C666 and HNE3 cells (*p* < 0.001, Figure 3a). These results indicate that BIRC5-206 knockdown facilitates molecular alterations associated with EMT progression. Conversely, overexpression of BIRC5-206 led to decreased expression of N-cadherin and Vimentin, and increased expression of E-cadherin and occludin (*p* < 0.001, Figure 3b), indicating that BIRC5-206 is involved in the regulation of EMT in NPC cells. Overall, our results demonstrate that BIRC5-206 knockdown promotes EMT progression in C666 and HNE3 cells, as evidenced by changes in the expression of key EMT markers.

### 3.3 BIRC5-206 knockdown promoted EMT *in vivo*

**Figure 4:**
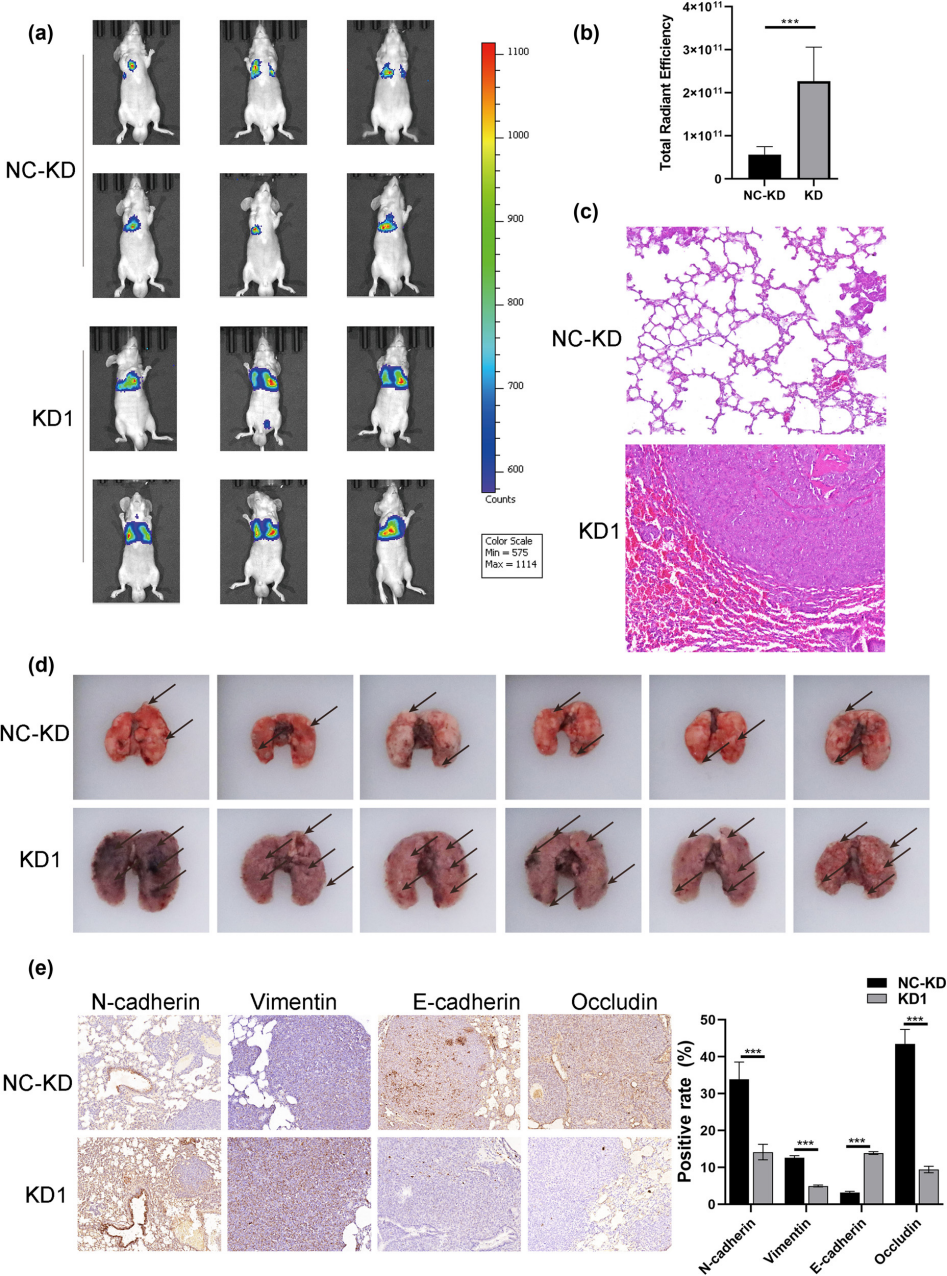
BIRC5-206 knockdown promotes EMT *in vivo*. 6-Week-old BALB/c mice were used to establish a mouse model of lung metastasis in NPC by intravenously injecting C666 cells with stable luciferase expression and BIRC5-206 knockdown. (a) and (b) *In vivo* tumor fluorescence images were acquired (*n* = 6). (c) Histological structure of lung tissues from each group was examined (HE staining, ×200). (d) After 6 weeks, lungs were harvested, and invasiveness foci were counted. Arrows indicate the metastatic foci in the lungs. (e) Immunohistochemistry for N-cadherin, Vimentin, E-cadherin, and occludin in lung tissues. Quantification of positive immunohistochemical staining.

We established a mouse model of lung invasiveness in NPC by intravenously injecting C666 cells with stable luciferase expression and BIRC5-206 knockdown. The tumor sizes in the BIRC5-206 knockdown group were notably larger than those in the control group, as demonstrated by *in vivo* bioluminescence imaging (Figure 4a and b). HE staining revealed that the lung tissue in the control group maintained normal and well-structured architecture, whereas the lung tissue in the BIRC5-206 knockdown group exhibited disrupted structures due to tumor cell infiltration (Figure 4c). Furthermore, there was a significant increase in the number of invasive foci in the lung tissues of the BIRC5-206 knockdown group compared to the control group (Figure 4d). Notably, IHC demonstrated a significant upregulation of N-cadherin and Vimentin expression levels, along with a notable downregulation of E-cadherin and occludin expression levels in the BIRC5-206 knockdown group (Figure 4e). These findings provide compelling evidence that silencing BIRC5-206 enhances the EMT process in NPC *in vivo*.

### 3.4 BIRC5-206 acted as a ceRNA for miR-145-5p in NPC

**Figure 5:**
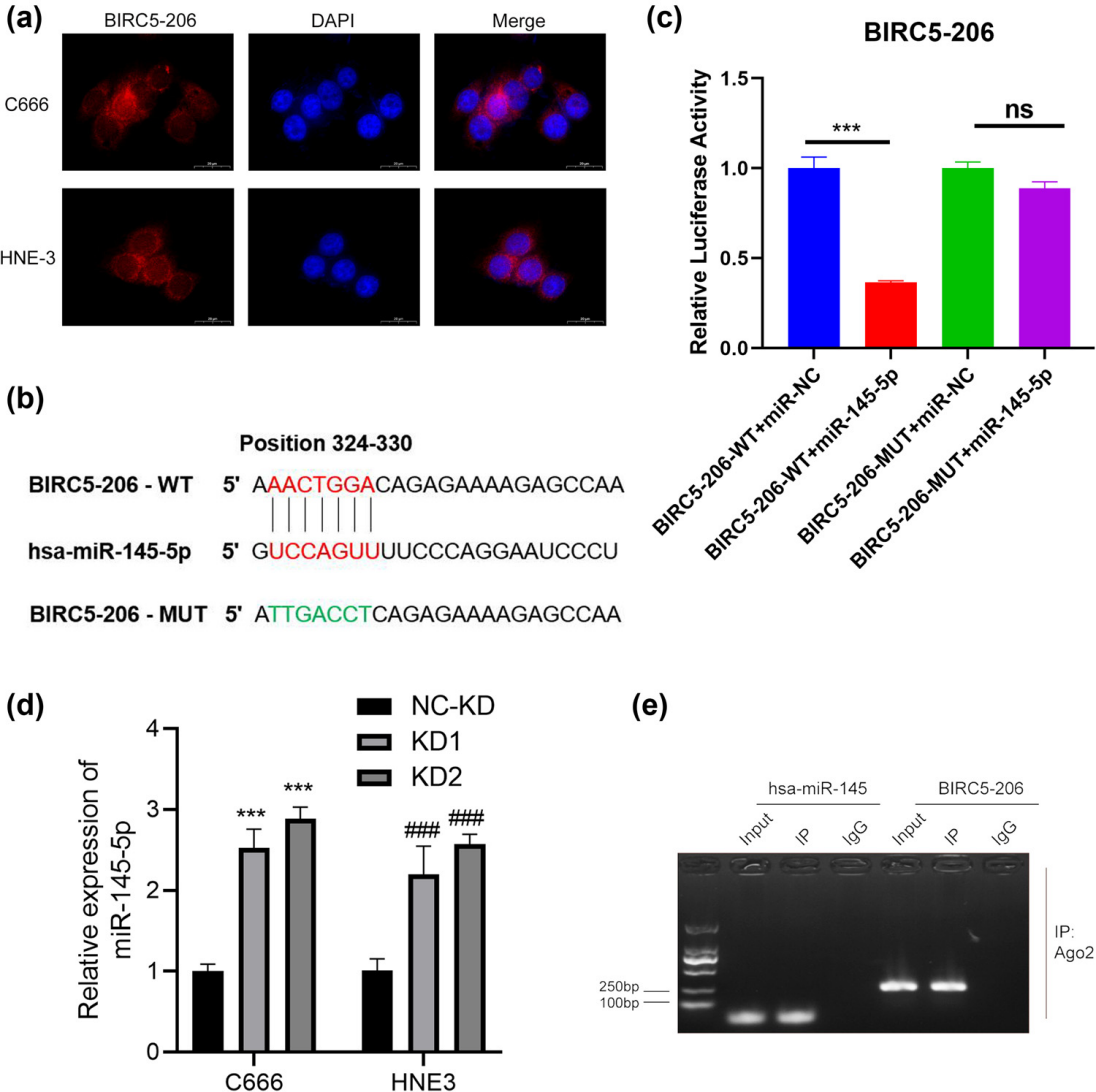
BIRC5-206 acted as a ceRNA for miR-145-5p in NPC. (a) Subcellular localization of BIRC5-206 in C666 and HNE3 cells was detected by FISH (scale bars: 20 μm). (b) The sequence of miR-145-5p binding sites in the 3′-UTR of BIRC5-206. The wild type (WT) and mutated (MUT) reporter constructs of the BIRC5-206 3′-UTR sequence are depicted in the schematic diagram in Figure 5b. (c) Luciferase reporter assay was performed to detect the relative luciferase activities of WT and MUT BIRC5-206 reporters. Luciferase activities were determined using the dual-luciferase reporter assay system. (d) miR-145-5p expression in BIRC5-206 knockdown C666 and HNE3 cells was detected by qPCR. (e) Ago2 RIP assay was conducted to detect the binding of BIRC5-206 and miR-145-5p in C666 cells. Results are expressed as means ± standard deviation (*n* = 3, ***p < 0.001).

To elucidate the molecular mechanisms underpinning BIRC5-206’s function in NPC cells, we first analyzed its cellular localization. We found that BIRC5-206 is in both the cytoplasm and nucleus, predominantly in the cytoplasm (Figure 5a). This indicates that BIRC5-206 may function as a competing endogenous RNA (ceRNA) in the cytoplasm, inhibiting EMT in NPC cells. By utilizing the Bibiserv database, we identified miR-145-5p as a potential target miRNA for BIRC5-206 (Figure 5b). To investigate the interaction between BIRC5-206 and miR-145-5p, we performed a dual-luciferase reporter assay. The results revealed that miR-145-5p significantly reduced the luciferase activity of the pmirGLO-BIRC5-206-WT construct, whereas it had no effect on the pmirGLO-BIRC5-206-MUT construct (Figure 5c). Moreover, the expression of miR-145-5p was significantly upregulated in C666 and HNE3 cells following BIRC5-206 knockdown, in comparison to the control group (Figure 5d). Furthermore, RIP analysis confirmed the binding of BIRC5-206 and miR-145-5p mRNAs to the Ago2 protein, further substantiating their interaction (Figure 5e). These findings have led us to identify miR-145-5p as a downstream target of BIRC5-206, and we intend to further explore its role in the molecular mechanisms of NPC.

### 3.5 BIRC5-206 downregulated the expression of CD40 through sponging miR-145-5p

**Figure 6:**
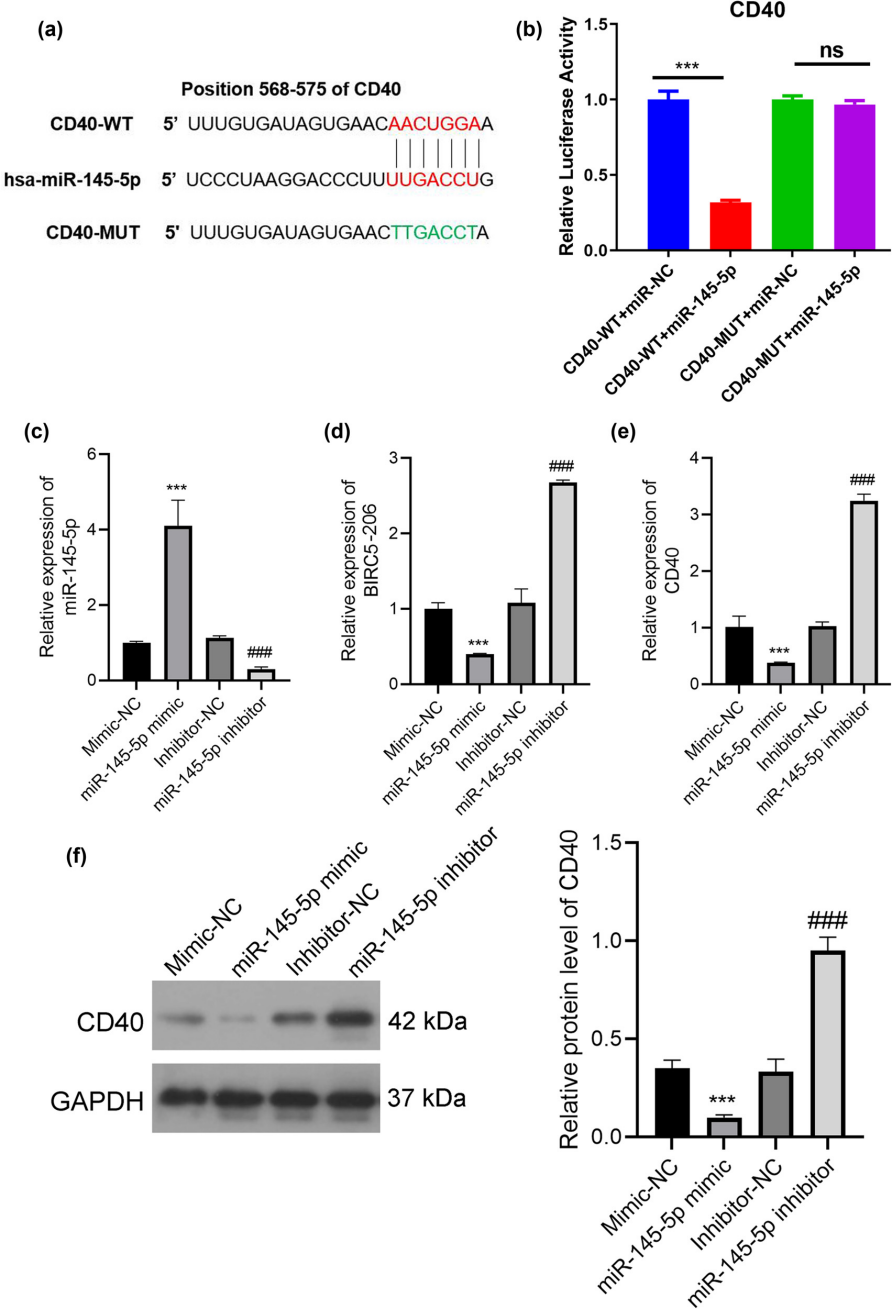
BIRC5-206 downregulates CD40 expression by sponging miR-145-5p. (a) The sequence of miR-145-5p binding sites in the 3′-UTR of CD40. The wild type (WT) and mutated (MUT) reporter constructs of the CD40 3′-UTR sequence are depicted in the schematic diagram in Figure 6a. (b) A luciferase reporter assay was performed to assess the relative luciferase activities of WT and MUT CD40 reporters. Luciferase activities were determined using the dual-luciferase reporter assay system. Expression of miR-145-5p (c), BIRC5-206 (d) and CD40 (e) in C666 cells following treatment with miR-145-5p mimic and inhibitor was detected by qPCR. (f) The protein level of CD40 in C666 cells post miR-145-5p mimic and inhibitor treatment was assessed by Western blot. Grayscale analysis of Western blot results was performed using ImageJ software. Results are expressed as means ± standard deviation (*n* = 3, ***p < 0.001).

**Figure 7:**
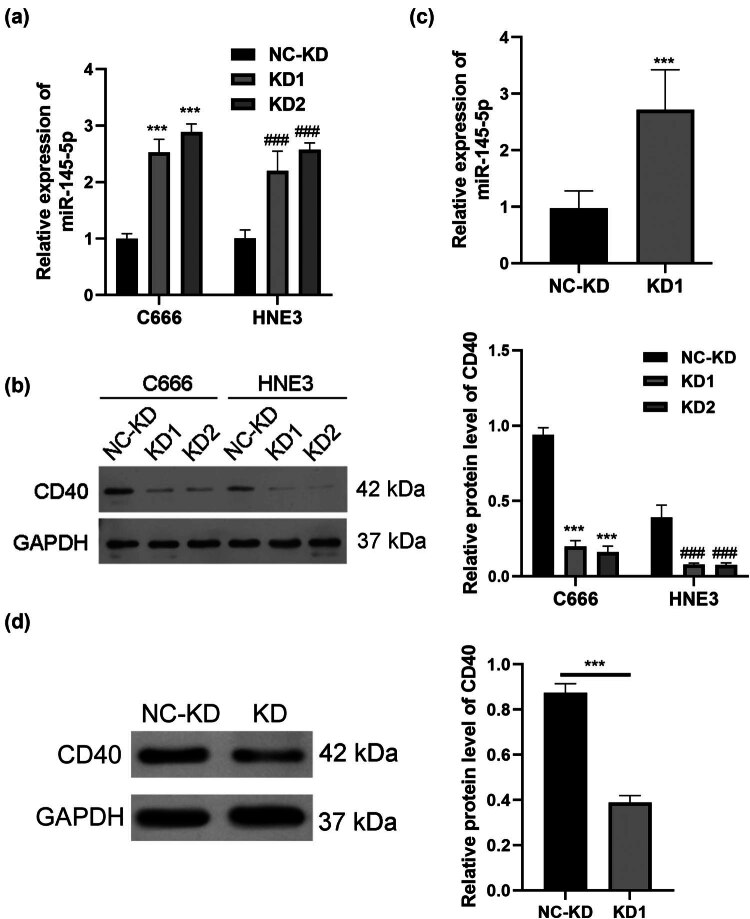
BIRC5-206 downregulates CD40 expression by sponging miR-145-5p. (a) CD40 mRNA expression in BIRC5-206 knockdown C666 and HNE3 cells was detected by qPCR. (b) CD40 protein expression in BIRC5-206 knockdown C666 and HNE3 cells was detected by Western blot. (c) miR-145-5p expression in lung tissues of a mouse model of lung metastasis in NPC was detected by qPCR. (d) CD40 protein level in lung tissues of the mouse model was detected by Western blot. Grayscale analysis of Western blot results was performed using ImageJ software. Results are expressed as means ± standard deviation (*n* = 3, ***p < 0.001).

To elucidate the impact of miR-145-5p on the mechanisms and functions of BIRC5-206, we utilized TargetScan to identify potential direct target genes for miR-145-5p. CD40 was identified as a notable candidate (Figure 6a). We then performed luciferase reporter assays to confirm the interaction between miR-145-5p and CD40. The results showed that miR-145-5p repressed the luciferase activity of the WT CD40 3′-UTR reporter plasmid, whereas this repression was not significant with the mutant (MUT) CD40 3′-UTR reporter plasmid (Figure 6b). Furthermore, qPCR analysis verified the effective modulation of miR-145-5p expression in C666 cells. Overexpression of miR-145-5p using a miR-145-5p mimic led to a significant reduction in BIRC5-206 expression, while inhibition of miR-145-5p resulted in a notable increase in BIRC5-206 expression (Figure 6c and d). Additionally, the miR-145-5p mimic group exhibited a significant decrease in both mRNA and protein expression of CD40, whereas the miR-145-5p inhibitor group exhibited a significant increase in these levels (Figure 6e and f). Furthermore, knockdown of BIRC5-206 in both C666 and HNE3 cells significantly inhibited CD40 mRNA and protein expression (Figure 7a and b). In the lung tissues of mice in a lung metastasis model, a marked increase in miR-145-5p expression (Figure 7c) and a substantial decrease in CD40 expression were observed following BIRC5-206 knockdown (Figure 7d).

**Figure 8:**
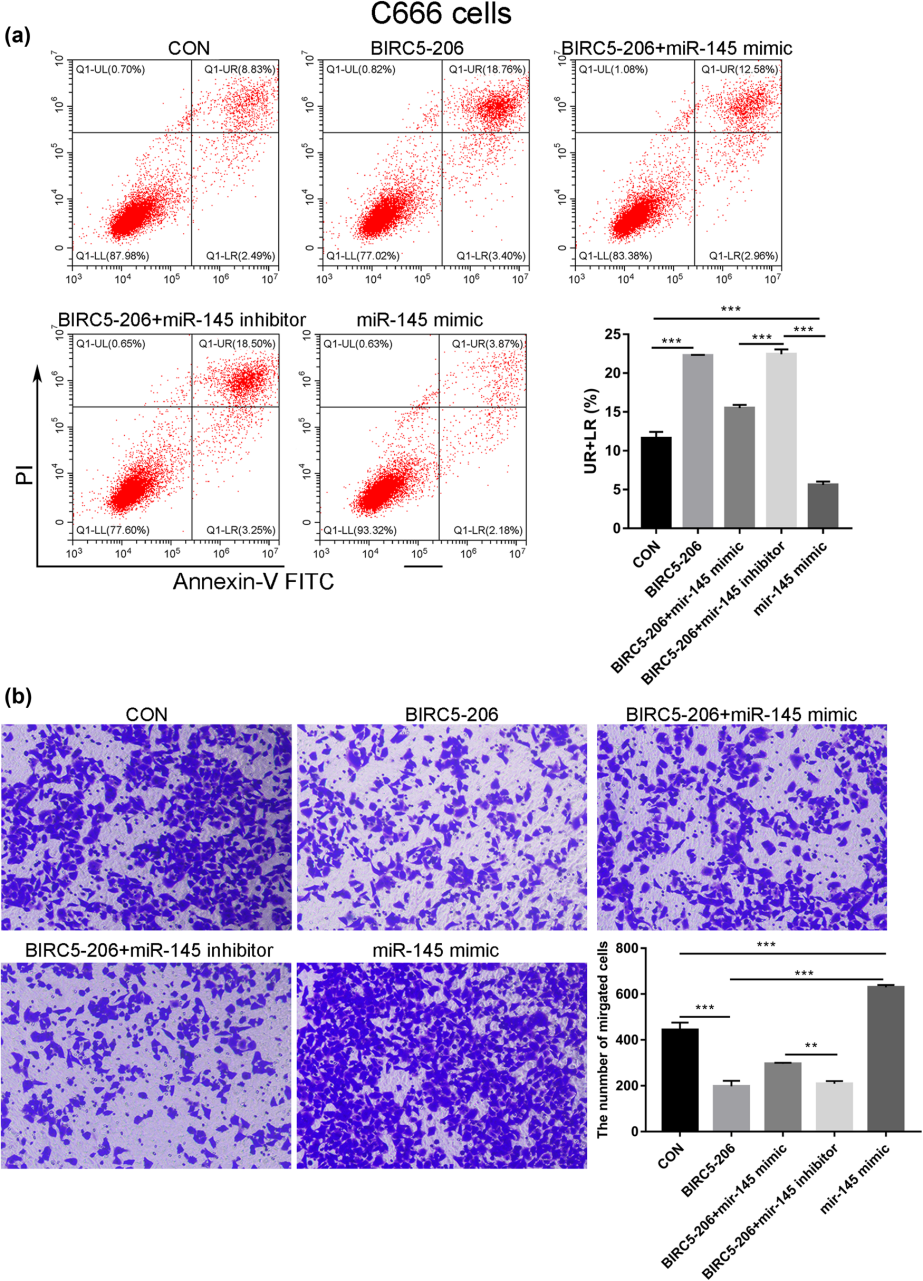
miR-145-5p partially mitigates the effect of BIRC5-206 overexpression on the apoptosis and invasion of C666 cells. BIRC5-206 overexpressing C666 cells were transfected with miR-145-5p mimic, mimic negative control (Mimic-NC), miR-145-5p inhibitor, and inhibitor negative control (Inhibitor-NC). (a) The apoptosis rate of C666 cells overexpressing BIRC5-206 and treated with miR-145-5p mimic/inhibitor was evaluated using Flow cytometry. (b) A Transwell invasion assay was conducted to assess the invasion ability of cells treated as described. Results are expressed as means ± standard deviation (*n* = 3, *p < 0.05, ***p < 0.001).

To further investigate the effects of BIRC5-206 and miR-145-5p in NPC cells, we treated C666 cells, which exhibit high BIRC5-206 expression, withmiR-145-5p mimic and inhibitor. The results indicated that compared to the control group, elevated BIRC5-206 expression significantly induced apoptosis in C666 cells, while miR-145-5p mimic markedly inhibited apoptosis (Figure 8a). Additionally, the miR-145-5p mimic partially counteracted the pro-apoptotic effect of high BIRC5-206 expression, whereas the miR-145-5p inhibitor substantially increased the proportion of apoptotic cells (Figure 8a). Moreover, high BIRC5-206 expression significantly reduced the invasion ability of C666 cells, whereas the miR-145-5p mimic markedly enhanced cell invasion (Figure 8b). The miR-145-5p mimic partially offset the inhibitory effect of high BIRC5-206 expression on cell invasion, while the miR-145-5p inhibitor further diminished the invasion capability of C666 cells (Figure 8b). These findings suggest that silencing of BIRC5-206 may facilitate the EMT process in NPC by sponging miR-145-5p, thereby leading to the downregulation of CD40.

## 4 Discussion

Tumor metastasis, characterized by the spread of malignant cells from the primary tumor to distant sites within the body, is a complex biological process [27]. It is one of the most severe aspects of cancer, significantly contributing to the reduced survival rates of cancer patients [28]. The 10-year survival rates for stage III and IV NPC cases are reported to be 74–79 and 46–56%, respectively, with stage IV cases exhibiting particularly poor prognosis [29]. Metastasis plays a crucial role in the unfavorable prognosis of advanced NPC, during which NPC cells can spread to nearby lymph nodes or distant organs. This spread poses challenges in completely eradicating the cancer cells and increases the risk of cancer recurrence [30]. However, the molecular mechanisms driving NPC metastasis remain incompletely understood. In our study, we have demonstrated that the downregulation of BIRC5-206 promotes NPC metastasis by targeting the miR-145-5p/CD40 axis.

As the smallest member of the IAP family, Survivin is upregulated in various human cancers and is positively associated with poor prognosis and drug resistance, establishing it as a significant tumor marker [31,32]. Due to its influential role in cancer cell proliferation and apoptosis, therapies targeting Survivin have been developed [14]. Different variants of Survivin, such as WT, 2B, and ΔEx3, are known to have distinct roles in cancer prognosis [33]. Elevated levels of Survivin ΔEx3 correlate with unfavorable clinical outcomes and prognosis in breast cancer [34]. In endometrial carcinomas, Survivin ΔEx3 has been linked to apoptosis, whereas Survivin WT is associated with cell proliferation [35]. The clinical and pathological significance of the Survivin 2B variant remains controversial; some studies associate it with disease progression, poor prognosis, and decreased survival, while others suggest it plays a role in disease remission [36–38].

Survivin proteins are highly expressed in NPC and are associated with poor overall survival, lymph node metastasis, local recurrence, distant metastasis, and a higher clinical stage of the disease [23,24]. Our previous research indicated that the noncoding splice variant of Survivin, BIRC5-206 (ENST00000589892.1), is expressed at low levels in NPC cells and promotes tumor progression in NPC, which is distinct from the expression patterns and roles of other known splicing variants of Survivin [25]. However, the specific role of BIRC5-206 in the tumorigenesis of NPC remains not fully understood. To delve deeper into the function of BIRC5-206 in NPC, we investigated its impact on apoptosis and invasion in NPC cell lines C666 and HNE3. Previous studies have shown that reduced Survivin expression suppresses proliferation and induces apoptosis in CNE-2 cells [24,39], while BIRC5-206 silencing decreases apoptosis and enhances invasion in both HONE1 and CNE-2 cells [25]. In this study, we confirmed that BIRC5-206 knockdown reduces apoptosis and increases invasion in C666 and HNE3 cells, aligning with its effects in HONE1 and CNE-2 cells. This observation contrasts with the roles of other Survivin splice variants in NPC progression [23–25]. Furthermore, we found that overexpressing BIRC5-206 elevates apoptosis and suppresses invasion in C666 and HNE3 cells. Additionally, in an NPC mouse model of lung invasiveness, the BIRC5-206 knockdown group exhibited a significant increase in invasive foci compared to the control group. These findings, both from *in vitro* and *in vivo* studies, indicate that reduced BIRC5-206 expression significantly contributes to NPC metastasis.

EMT, a process where cells transition from an epithelial to a mesenchymal state, is critical in promoting tumor metastasis [40]. This transition endows cancer cells with enhanced migratory and invasive capabilities, enabling them to detach from the primary tumor and migrate through the bloodstream or lymphatic system to establish metastases [40]. EMT involves molecular alterations in epithelial cells, characterized by the loss of cell–cell adhesion and the acquisition of mesenchymal traits [7]. These alterations include the downregulating epithelial markers like E-cadherin and the upregulating mesenchymal markers such as N-cadherin and Vimentin [8–10]. In our study, we found that knocking down BIRC5-206 significantly increased N-cadherin and Vimentin protein levels, while reducing the expression of E-cadherin and occludin in both C666 and HNE3 cells. In contrast, overexpressing BIRC5-206 produced the opposite effect. Additionally, in the NPC mouse model of lung invasiveness, significant upregulation of N-cadherin and Vimentin, along with downregulation of E-cadherin and occludin, was observed in the lung tissues of the BIRC5-206 knockdown group. These results provide compelling evidence that silencing BIRC5-206 promotes EMT in NPC, both *in vitro* and *in vivo*, which may contribute to the enhanced metastatic potential of NPC following BIRC5-206 knockdown.

Long noncoding RNA (lncRNA) is a class of transcripts typically exceeding 200 nucleotides in length, without the capacity to encode proteins [41]. lncRNAs demonstrate a broader structural and functional diversity compared to protein-coding mRNAs. They regulate gene expression through diverse mechanisms, including chromatin modification, transcriptional control, and post-transcriptional regulation [42]. In tumors, dysregulated lncRNAs are intimately linked with tumorigenesis and tumor progression [43]. They can function as tumor suppressors or oncogenes, influencing various biological processes such as cell proliferation, apoptosis, invasion, metastasis, and angiogenesis [43,44]. Several lncRNAs, including LINC00460 [45], NEAT1 [46], LINC00930 [47], and DIAPH1-AS1 [48], have been identified as key players in NPC. One regulatory mechanism of lncRNAs is their role as ceRNAs, where they bind to miRNAs, reducing the miRNA-mediated regulation of target genes [49]. For example, the NEAT1/let-7a-5p axis regulates cisplatin resistance in NPC [46], LINC00460 upregulates IL6 by sequestering miR-149-5p to promote NPC development [45], and lncRNA SNHG1 acts as a ceRNA to mitigate the downregulation of NUAK1 by miR-145a-5p in NPC cells [50]. In this study, bioinformatics analysis predicted miR-145-5p as a direct target of BIRC5-206, a finding further substantiated by luciferase reporter and RIP assays. We also noted a negative correlation between the expression levels of BIRC5-206 and miR-145-5p both *in vitro* and *in vivo*. Functional experiments with miR-145-5p mimic and inhibitor partially counteracted and enhanced, respectively, the effects of high BIRC5-206 expression on cell apoptosis and invasion. Thus, we propose that BIRC5-206 downregulation leads to miR-145-5p upregulation, thereby facilitating the progression of EMT in NPC cells.

miR-145 is highly expressed in various malignancies and plays a significant role in cancer initiation and therapeutic resistance [51]. It functions in cancer by regulating downstream molecules or by being regulated by upstream lncRNA and circRNA, which act as ceRNA. For instance, the lncRNA ROR/miR-145-5p axis promotes osteoblast proliferation in osteoporosis [52], and silencing of lncRNA MALAT1 enhances erastin-induced ferroptosis in endometriosis via the miR-145-5p/MUC1 signaling pathway [53]. Furthermore, hsa_circ_0001955/miR-145-5p augments the proliferation and invasion of HCC cells by targeting NRAS [54]. In our study, we identified CD40 as a crucial effector of miR-145-5p. CD40, a member of the tumor necrosis factor receptor superfamily, is present on the surface of T cells [55]. The interaction between CD40 and CD154 can activate T cells, eliciting an immune response against tumors [56]. Decreased CD40 expression has been linked to poor responses to immune checkpoint blockade therapy and unfavorable clinical outcomes [57]. Through bioinformatics analysis, we predicted CD40 as a direct target of miR-145-5p, a finding that was confirmed by luciferase reporter assays. The overexpression of the miR-145-5p mimic inhibited CD40 protein levels while inhibiting miR-145-5p significantly increased CD40 protein levels. We also demonstrated that silencing BIRC5-206 led to decreased CD40 protein levels both *in vivo* and *in vitro*. CD40 has been identified as a novel partial EMT-specific marker [58]. It has been observed that CD40 expression is upregulated in cells undergoing partial EMT but decreased in fully transitioned EMT cells. Additionally, the interactions between CD40 and CD40L play a crucial role during EMT and aortic remodeling [59]. Based on these results, we hypothesize that BIRC5-206 downregulation decreases CD40 expression through direct interaction with miR-145-5p, thereby facilitating NPC metastasis by promoting EMT progression. Our findings indicate that BIRC5-206 is closely associated with NPC progression, suggesting its potential as a biomarker with significant prognostic and therapeutic value.

## 5 Conclusion

The downregulation of BIRC5-206 facilitates the EMT process in NPC by functioning as a sponge for miR-145-5p, which consequently leads to the downregulation of CD40. These significant findings highlight BIRC5-206 as a promising novel prognostic biomarker and potential therapeutic target for the diagnosis and treatment of NPC.
